# Non-intubated vs. intubated video-assisted thoracoscopic surgery for the treatment of thoracic diseases: a systematic review and meta-analysis of propensity score-matched cohorts

**DOI:** 10.3389/fsurg.2025.1661466

**Published:** 2025-09-26

**Authors:** Tao Luo, Yue Zhang, Bin-Wen Xu, Cheng-Cheng Zhang, Li-Wen Zhang, Xin-Qiang Ran, Mao-Yong Fu

**Affiliations:** Department of Thoracic Surgery, Affiliated Hospital of North Sichuan Medical College, Nanchong, China

**Keywords:** thoracoscopic surgery, non-intubated anesthesia, meta-analysis, spontaneous breathing, intubated anesthesia

## Abstract

**Background:**

Video-assisted thoracic surgery (VATS) is commonly conducted under general anesthesia with tracheal intubation, which can lead to intubation-related complications. Consequently, non-intubated video-assisted thoracoscopic surgery (NIVATS) has gained increasing attention. However, debates persist regarding its safety and efficacy. This study aims to compare the safety and efficacy of NIVATS vs. intubated VATS (IVATS) in thoracic surgery.

**Methods:**

Relevant literature published up to October 2024 was collected from the Cochrane, PubMed, and Embase databases based on predefined inclusion criteria. Two reviewers independently screened these studies and extracted the pertinent data. After assessing the quality of the included studies, a meta-analysis was performed using Review Manager 5.3. Fixed-effects or random-effects models were utilized to synthesize the combined data.

**Results:**

Compared with IVATS, NIVATS demonstrated shorter operative time [weighted mean difference (WMD) = −23.33 min; 95% confidence interval (CI), −33.62 to −13.04; *P* < 0.01], reduced anesthesia time (WMD = −29.10 min; 95% CI, −48.16 to −10.04; *P* < 0.01), shorter length of hospital stay (WMD = −0.46 days; 95% CI, −0.82 to −0.10; *P* = 0.01), and decreased chest tube drainage duration (WMD = −0.40 days; 95% CI, −0.71 to −0.09; *P* = 0.01). However, regarding the incidence of postoperative complications, contrary to previous meta-analyses, the NIVATS group exhibited a higher rate of postoperative complications (odds ratio = 1.87; 95% CI, 1.17–2.98; *P* = 0.008). No significant differences were observed between the groups in terms of intraoperative bleeding, the number of lymph nodes harvested, the number of N1 lymph nodes, the number of N2 lymph nodes, or postoperative length of hospital stay.

**Conclusion:**

Although there were no significant differences between the two anesthesia methods concerning postoperative length of hospital stay, lymph node dissection, or intraoperative bleeding, NIVATS significantly reduced operative time, anesthesia time, length of hospital stay, and chest tube drainage duration. For highly selected patients, NIVATS may offer additional advantages. However, the increased postoperative complication rate associated with NIVATS compared with IVATS warrants further large, well-designed randomized trials.

## Introduction

Intubated video-assisted thoracic surgery (IVATS) is the standard treatment for numerous thoracic diseases, including lung cancer, pulmonary nodules, bullous emphysema, and palmar hyperhidrosis (1–9). It is favored by most thoracic surgeons due to its minimally invasive nature, stable surgical field, and adequate operative space (10). However, tracheal intubation under general anesthesia has been associated with several postoperative complications, such as airway injury, respiratory-induced lung injury, postoperative nausea and vomiting, diaphragmatic dysfunction, pulmonary inflammation, and postoperative sore throat (11–18). To mitigate these complications and in pursuit of less invasive surgical strategies, non-intubated video-assisted thoracoscopic surgery (NIVATS) has gradually emerged as a promising alternative in thoracic surgery.

Although network meta-analyses on this topic have been conducted (19–22), these analyses included non-controlled observational studies that could introduce confounding factors and did not incorporate recently published research. Therefore, we conducted an up-to-date systematic review and meta-analysis of high-quality propensity score-matched (PSM) studies to compare the safety and efficacy between NIVATS and IVATS.

## Methods

### Search strategy

This review was registered with PROSPERO on 2 December 2024 (registration number CRD42024621145). A systematic and comprehensive computer-based search was conducted using the Cochrane, PubMed, and Embase databases, employing the search strategy “(((non-intubated) OR (spontaneous breathing anesthesia)) OR (spontaneous ventilation anesthesia)) OR (awake anesthesia)) AND (((video-assisted transthoracic surgery) OR (VATS)) OR (thoracoscope)).” Relevant high-quality studies that utilized propensity score matching and were published up to October 2024 were selected without restrictions on publication year or country. In addition, the reference lists of original articles and review articles were manually screened to identify studies not captured in the database search.

### Inclusion and exclusion criteria

Studies were included based on the following criteria: (1) studies comparing NIVATS with intubated video-assisted thoracoscopic surgery (IVATS) in thoracic surgery; (2) studies providing sufficient data to calculate mean differences (MDs) or odds ratios (ORs); (3) studies in which both patient groups underwent propensity score matching to reduce the influence of confounding factors; and (4) in cases of duplicate publications, the most recent study was selected. The exclusion criteria were as follows: (1) studies that did not compare non-intubated VATS with intubated VATS; (2) studies in which the surgical methods differed between intubated and non-intubated patients; (3) reviews, letters, editorials, expert opinions, case reports, and animal experiments; and (4) studies from which relevant data could not be extracted.

### Data extraction

Data screening was independently conducted by two authors, who extracted relevant data from studies that met the inclusion criteria. In cases of discrepancies during data selection, the two authors resolved them through consultation, and unresolved differences were ultimately decided by the corresponding author. The extracted data included the following: first author, year of publication, study design, number of study subjects, incidence of postoperative complications, operative time, anesthesia time, length of hospital stay, number of lymph nodes dissected intraoperatively, number of N1 lymph nodes dissected, number of N2 lymph nodes dissected, postoperative length of stay, intraoperative blood loss, and chest tube placement time (postoperative complications included atelectasis, air leakage, pulmonary infection, etc.).

### Assessment of quality

The quality of all included studies was assessed using the Newcastle–Ottawa scale (NOS) (23, 24), based on three factors: selection of study participants, comparability between groups, and measurement of exposure. Each study was scored from 0 to 9 points according to these three parameters, with studies scoring ≥6 points classified as high-quality and those scoring below 6 points classified as low-quality studies.

### Statistical analysis

The meta-analysis was conducted using Review Manager 5 software (RevMan 5.3, Cochrane Community, London, UK). Statistical heterogeneity was assessed using Higgins' *I*^2^, which represents the percentage of total variation across studies attributable to heterogeneity. When *I*^2^ was <50%, a fixed-effect model (Mantel–Haenszel method) was used to pool homogeneous studies; otherwise, a random-effects model (DerSimonian–Laird) was employed. For quantitative data, the effect measures were MDs with 95% confidence intervals (CI), and for qualitative data, the effect measures were OR with 95% CI. A *P*-value of <0.05 was considered statistically significant. The median and interquartile range of continuous variables were converted to mean and standard deviation (SD) using the sample mean estimation method (25) and the SD estimation method (26), respectively, via an online tool (http://www.comp.hkbu.edu.hk/∼xwan/median2mean.html).

### Sensitivity analysis

We utilized the leave-one-out method to sequentially exclude each study from the pooled effect to evaluate the robustness of the estimates. Furthermore, we assessed robustness based on the size of the study cohorts (excluding studies with fewer than 100 patients), as this may contribute to heterogeneity. However, sensitivity analysis could not be performed for three or fewer studies.

### Publication bias

When 10 or fewer studies were included, the tests lacked sufficient power. Therefore, we did not conduct further publication bias analyses (27, 28).

## Results

### Baseline characteristics

**Figure 1:**
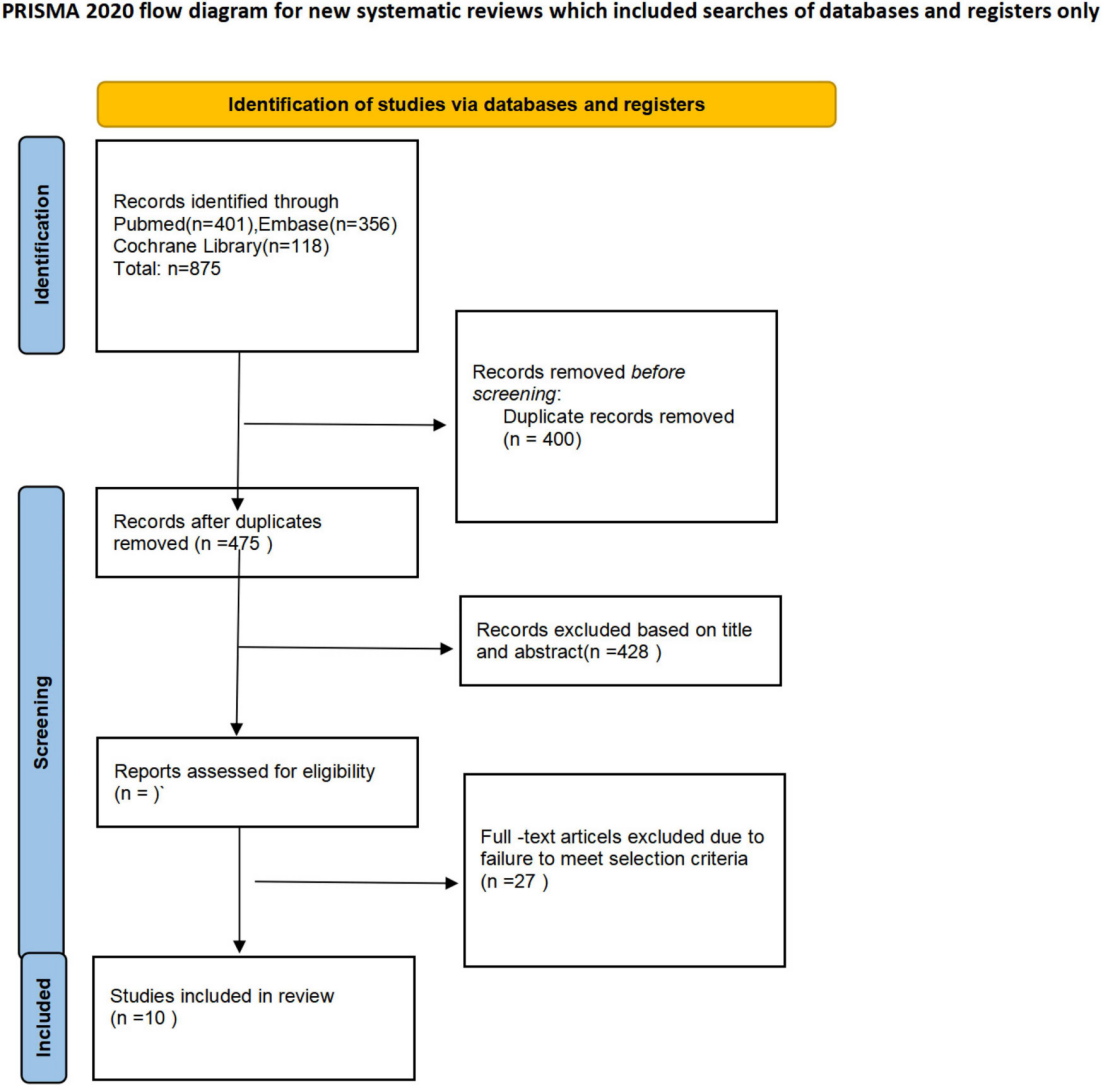
Search strategy.

Initially, a total of 875 articles were retrieved through the literature search. After removing 400 duplicates, 475 studies remained for screening. Of these, 428 papers (including conference abstracts, letters, case reports, or related studies) were excluded from the screening process. The full texts of the remaining 37 studies were further assessed, leading to the exclusion of 27 papers due to factors such as lack of data specificity, non-adult subjects, and incorrect interventions. Ultimately, 10 studies were accepted and included in the meta-analysis. These studies compared the safety and efficacy of NIVATS vs. intubated video-assisted thoracoscopic surgery (IVATS) for the treatment of thoracic diseases (29–38). Table 1 presents the main data extracted from the included studies, and Table 2 summarizes their main characteristics. The 10 studies encompassed a total of 1,882 patients: 1,014 underwent IVATS, and 868 underwent NIVATS. Figure 1 illustrates the search strategy.

**Table 1:** Main data extracted from the studies.

Reference	The operative time (min)		Anesthesia time (min)		Intraoperative bleeding volume (mL)		Days of chest tube use (days)		Postoperative hospital stay (days)	
	Non-intubated	Intubated	Non-intubated	Intubated	Non-intubated	Intubated	Non-intubated	Intubated	Non-intubated	Intubated
Zheng et al. (29)	158.56 ± 40.09	172.06 ± 61.75	247.4 ± 62.49	256.7 ± 58.52	78.88 ± 80.25	109.932 ± 180.86	4.03 ± 2.19	4.29 ± 3.02	NR	NR
Wang et al. (30)	166.2 ± 102.6	170.1 ± 83.4	226.3 ± 79.8	238.5 ± 66.2	61.5 ± 165.1	82.2 ± 116.9	NR	NR	NR	NR
Deng et al. (31)	NR	NR	185.2766 ± 110.2852	210.7105 ± 94.0895	NR	NR	3.3553 ± 3.8561	3.7105 ± 1.5425	7.3553 ± 3.8561	6.3553 ± 3.8561
Chen et al. (32)	97.5 ± 39.7	105.8 ± 54.8	NR	NR	15.2 ± 49.6	21.2 ± 77.1	2.8 ± 4	3.4 ± 3.9	4.8 ± 4.2	6.6 ± 8.5
Pathonsamit et al. (33)	65.3063 ± 41.9344	116.0729 ± 81.9627	NR	NR	NR	NR	NR	NR	NR	NR
Wu et al. (34)	122 ± 44	142 ± 56	213 ± 57	233 ± 67	49 ± 51	48 ± 38	4 ± 3	4 ± 2	NR	NR
Udelsman et al. (35)	70.7646 ± 41.6706	117.2427 ± 59.2042	NR	NR	NR	NR	NR	NR	NR	NR
Lan et al. (36)	175.63 ± 55.67	217.64 ± 59.71	250.63 ± 55.67	301.87 ± 63.69	67.5819 ± 67.5406	152.7458 ± 187.6128	NR	NR	NR	NR
Liu et al. (37)	177.8 ± 43.0	182.0 ± 55.5	NR	NR	124.4 ± 115.2	142.6 ± 221.6	3.2 ± 2.6	3.5 ± 2.4	7.4 ± 2	8.6 ± 4.1
Elkhouly et al. (38)	37 ± 19	61 ± 23	30 ± 23	81 ± 49	NR	NR	2.9 ± 1.7	3.8 ± 2.5	NR	NR
Reference	Lymph node number		Hospitalization (day)		Number of N1 stations		Number of N2 stations		All complications	
	Non-intubated	Intubated	Non-intubated	Intubated	Non-intubated	Intubated	Non-intubated	Intubated	Non-intubated	Intubated
Zheng et al. (29)	4.64 ± 3.9	4.78 ± 3.49	NR	NR	1.46 ± 1.12	1.47 ± 0.99	10.91 ± 8.35	12.04 ± 7.83	NR	NR
Wang et al. (30)	5.3 ± 7.5	4.4 ± 7.4	17.6 ± 7.6	17.2 ± 6.9	2.1 ± 4.2	1.6 ± 3	3.1 ± 5.1	2.8 ± 5.2	NR	NR
Deng et al. (31)	NR	NR	NR	NR	NR	NR	NR	NR	NR	NR
Chen et al. (32)	6.4 ± 5.6	8.3 ± 7	NR	NR	NR	NR	NR	NR	NR	NR
Pathonsamit et al. (33)	NR	NR	5.3538 ± 2.2873	7.0613 ± 3.8122	NR	NR	NR	NR	NR	NR
Wu et al. (34)	NR	NR	15 ± 8	16 ± 7	3 ± 4	2 ± 5	6 ± 9	3 ± 6	6/48	2/48
Udelsman et al. (35)	NR	NR	2 ± 1.5153	2.3515 ± 0.7494	NR	NR	NR	NR	NR	NR
Lan et al. (36)	NR	NR	NR	NR	NR	NR	NR	NR	41/119	24/119
Liu et al. (37)	17.2 ± 9.1	15.7 ± 9.5	NR	NR	NR	NR	NR	NR	12/116	10/116
Elkhouly et al. (38)	NR	NR	NR	NR	NR	NR	NR	NR	NR	NR

Data not reported are indicated as NR. Continuous variables are presented as mean ± standard deviation [operative time (min), anesthesia time (min), intraoperative bleeding volume (mL), days of chest tube use (day), postoperative hospital stay (day), lymph node number, hospitalization (day), number of N1 stations, number of N2 stations]. Binary variables are presented as the number of patients with complications/the number of patients without complications (all complications).

**Table 2:** Characteristics of the studies included in the meta-analysis.

Study	Year	Country	Study design	Disease	Sample size (total/intubated/non-intubated)	Quality assessment NOS
Zheng et al.	2021	China	Retrospective	Non-small cell lung cancer	400/200/200	8
Wang et al.	2022	China	Retrospective	Non-small cell lung cancer	160/80/80	8
Deng et al.	2022	China	Retrospective	Variety	74/37/37	7
Chen et al.	2022	China	Retrospective	Non-small cell lung cancer	237/158/79	7
Pathonsamit et al.	2022	Thailand	Retrospective	Variety	104/52/52	8
Wu et al.	2020	China	Retrospective	Non-small cell lung cancer	96/48/48	8
Udelsman et al.	2024	United States	Retrospective	Variety	201/134/67	7
Lan et al.	2018	China	Retrospective	Variety	238/119/119	7
Liu et al.	2016	China	Retrospective	Non-small cell lung cancer	232/116/116	8
Elkhouly et al.	2022	Egypt	Retrospective	Pulmonary bulla	140/70/70	7

NOS, Newcastle–Ottawa scale.

### Sensitivity analysis and assessment of quality

In this meta-analysis, certain outcomes (operative time, anesthesia time, intraoperative blood loss, and number of lymph nodes dissected) exhibited high heterogeneity. To achieve stable and reliable conclusions, sensitivity analyses were performed on these target parameters. Utilizing the leave-one-out method to recalculate the sample size, we found that after excluding one study (36), the heterogeneity in postoperative blood loss was significantly reduced (Figure 4C). Similarly, excluding another study (32) notably decreased the heterogeneity in the number of lymph nodes dissected (Figure 4D). Despite these adjustments, the results continued to show no significant differences between the NIVATS and IVATS groups, confirming the stability of the findings. The remaining outcomes also remained stable. Quality assessments of the 10 included studies demonstrated that they exhibited high methodological quality.

### Meta-analysis results

**Figure 2:**
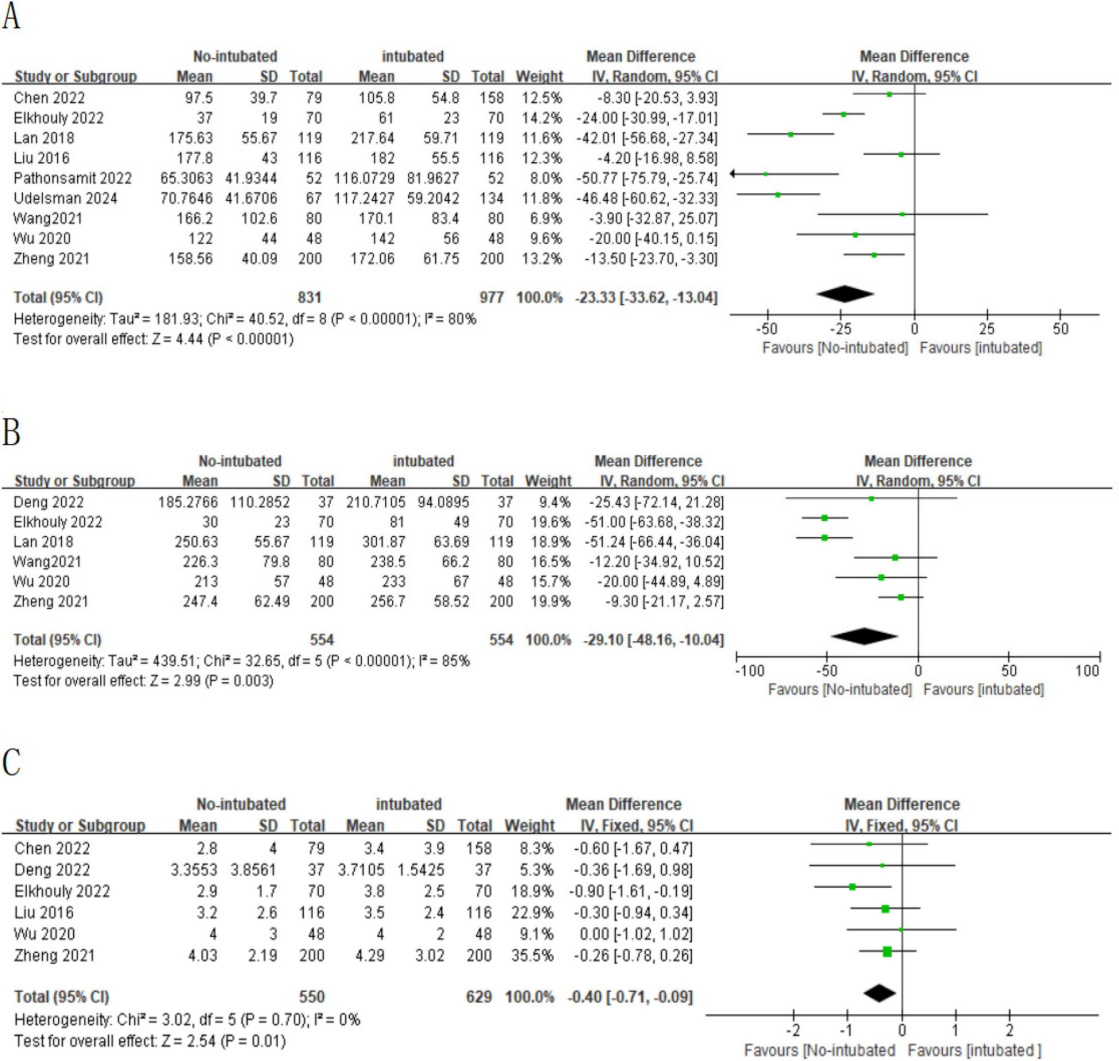
(**A**) Forest plot of operative time comparing the non-intubated group and the intubated group. (**B**) Forest plot of anesthesia time comparing the non-intubated group and the intubated group. (**C**) Forest plot of chest tube drainage duration comparing the non-intubated group and the intubated group. CI, confidence interval; IV, inverse variance; SD, standard deviation.

**Figure 3:**
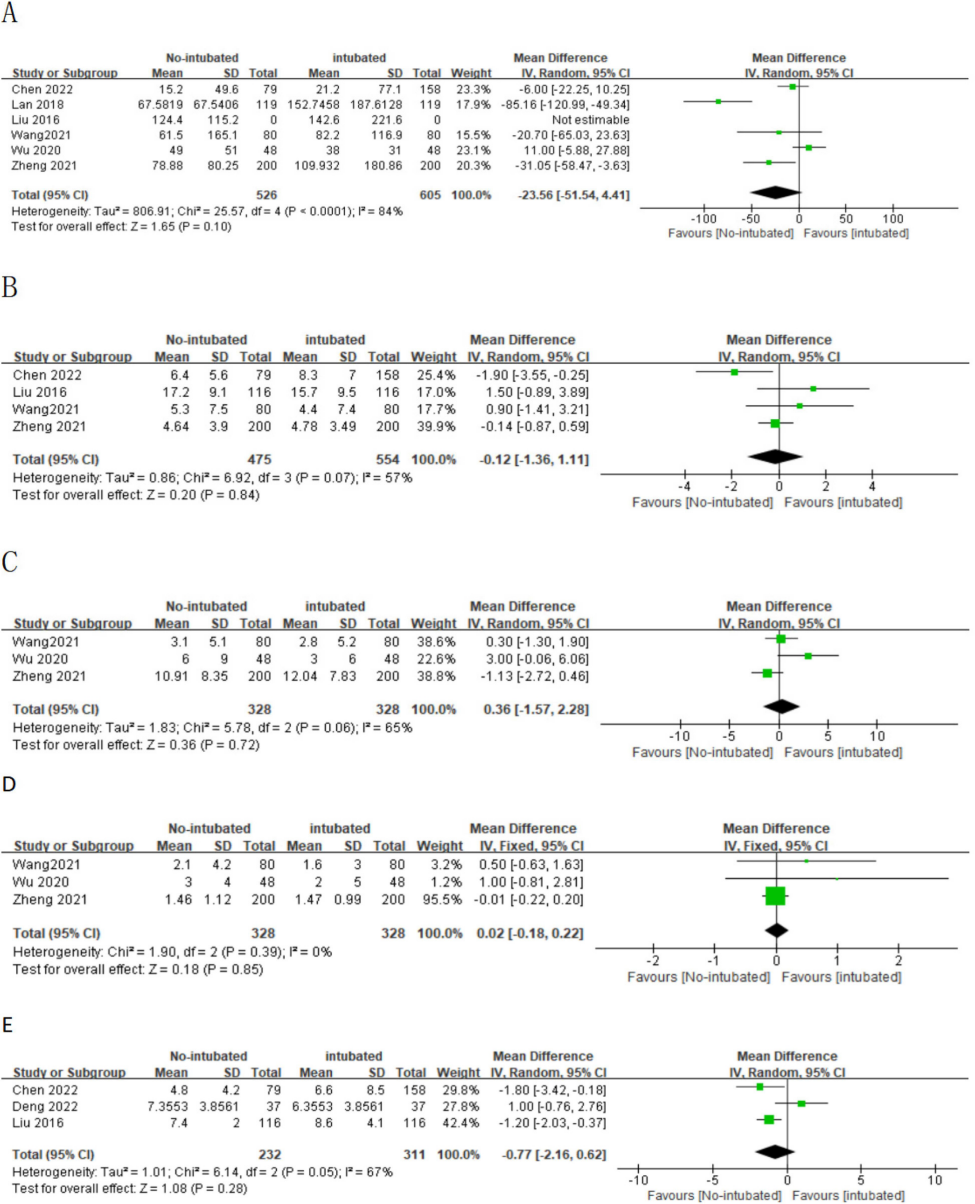
(**A**) Forest plot of intraoperative blood loss comparing the non-intubated group and the intubated group. (**B**) Forest plot of lymph node dissection comparing the non-intubated group and the intubated group. (**C**) Forest plot of N2 lymph node dissection comparing the non-intubated group and the intubated group. (**D**) Forest plot of N1 lymph node dissection comparing the non-intubated group and the intubated group. (**E**) Forest plot of postoperative hospital stay comparing the non-intubated group and the intubated group. CI, confidence interval; IV, inverse variance; SD, standard deviation.

**Figure 4:**
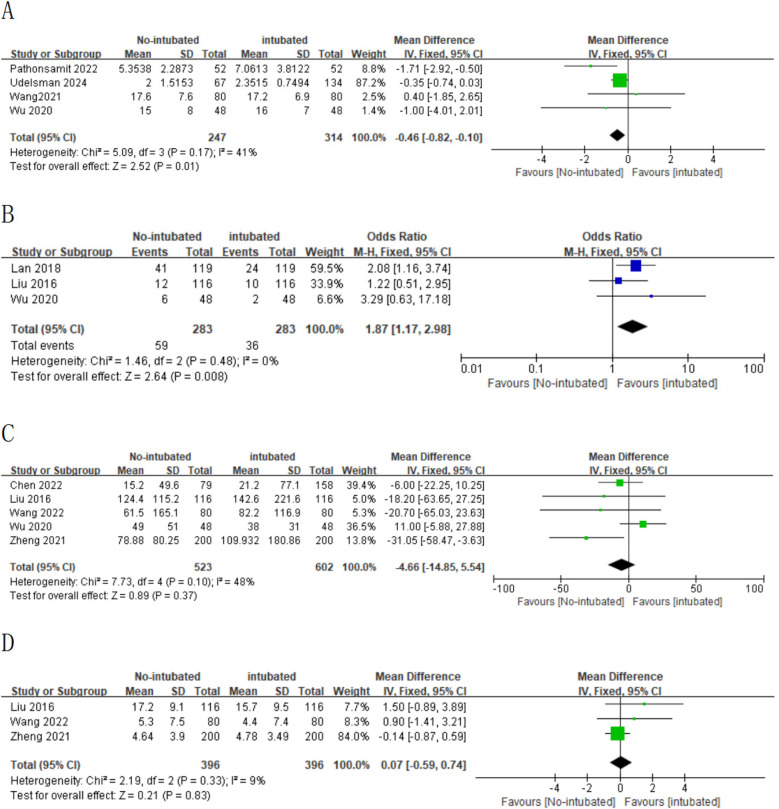
(**A**) Forest plot of length of hospital stay comparing the non-intubated group and the intubated group. (**B**) Forest plot of postoperative complication rates comparing the non-intubated group and the intubated group. (**C**) Forest plot of intraoperative blood loss comparing the non-intubated group and the intubated group [excluding Lan et al. (2018) study]. (**D**) Forest plot of lymph node dissection comparing the non-intubated group and the intubated group [excluding Chen et al. (2022) study].

A meta-analysis was conducted on 10 studies that met the inclusion criteria to compare the feasibility and safety of NIVATS vs. IVATS. Among them, nine studies compared the operative time between NIVATS and IVATS, revealing that NIVATS significantly reduced the operative time compared with IVATS [weighted mean difference (WMD) = −23.33 min; 95% CI), −33.62 to −13.04; *P* < 0.01] (Figure 2A). Based on six studies reporting anesthesia time, NIVATS was associated with a significant reduction in anesthesia time compared with IVATS (WMD = −29.10 min; 95% CI, −48.16 to −10.04; *P* < 0.01) (Figure 2B). Regarding chest tube drainage duration, six studies indicated that the NIVATS group had a significantly shorter chest tube drainage time (WMD = −0.40 days; 95% CI, −0.71 to −0.09; *P* = 0.01) (Figure 2C). Six studies reported on intraoperative blood loss, showing no significant difference between NIVATS and IVATS (WMD = −23.56 mL; 95% CI, −51.54 to 4.41; *P* = 0.1) (Figure 3A). In addition, four studies examined the number of lymph nodes dissected intraoperatively, with no significant difference observed (WMD = −0.12 nodes; 95% CI, −1.36 to 1.11; *P* = 0.84) (Figure 3B). Three studies reported the number of N2 lymph nodes, and no significant difference was found (WMD = −0.36 nodes; 95% CI, −1.57 to 2.28; *P* = 0.72) (Figure 3C). Similarly, for the number of N1 lymph nodes, three studies showed no significant difference (WMD = 0.02 nodes; 95% CI, −0.18 to 0.22; *P* = 0.85) (Figure 3D). Postoperative hospital stay was reported in six studies, with no significant difference between the NIVATS and IVATS groups (WMD = −0.77 days; 95% CI, −2.16 to 0.62; *P* = 0.85) (Figure 3E). However, four studies reported on the length of hospital stay, demonstrating that NIVATS significantly reduced the length of hospital stay compared with IVATS (WMD = −0.46 days; 95% CI, −0.82 to −0.10; *P* = 0.01) (Figure 4A). Furthermore, the incidence of postoperative complications was significantly higher in the NIVATS group compared with the IVATS group based on a meta-analysis of three studies (OR = 1.87; 95% CI, 1.17 to 2.98; *P* = 0.008) (Figure 4B).

### Subgroup analysis

Outcomes included at least in three studies were further evaluated by subgroup analysis.

### NIVATS (non-small cell lung cancer) vs. IVATS (non-small cell lung cancer)

**Figure 5:**
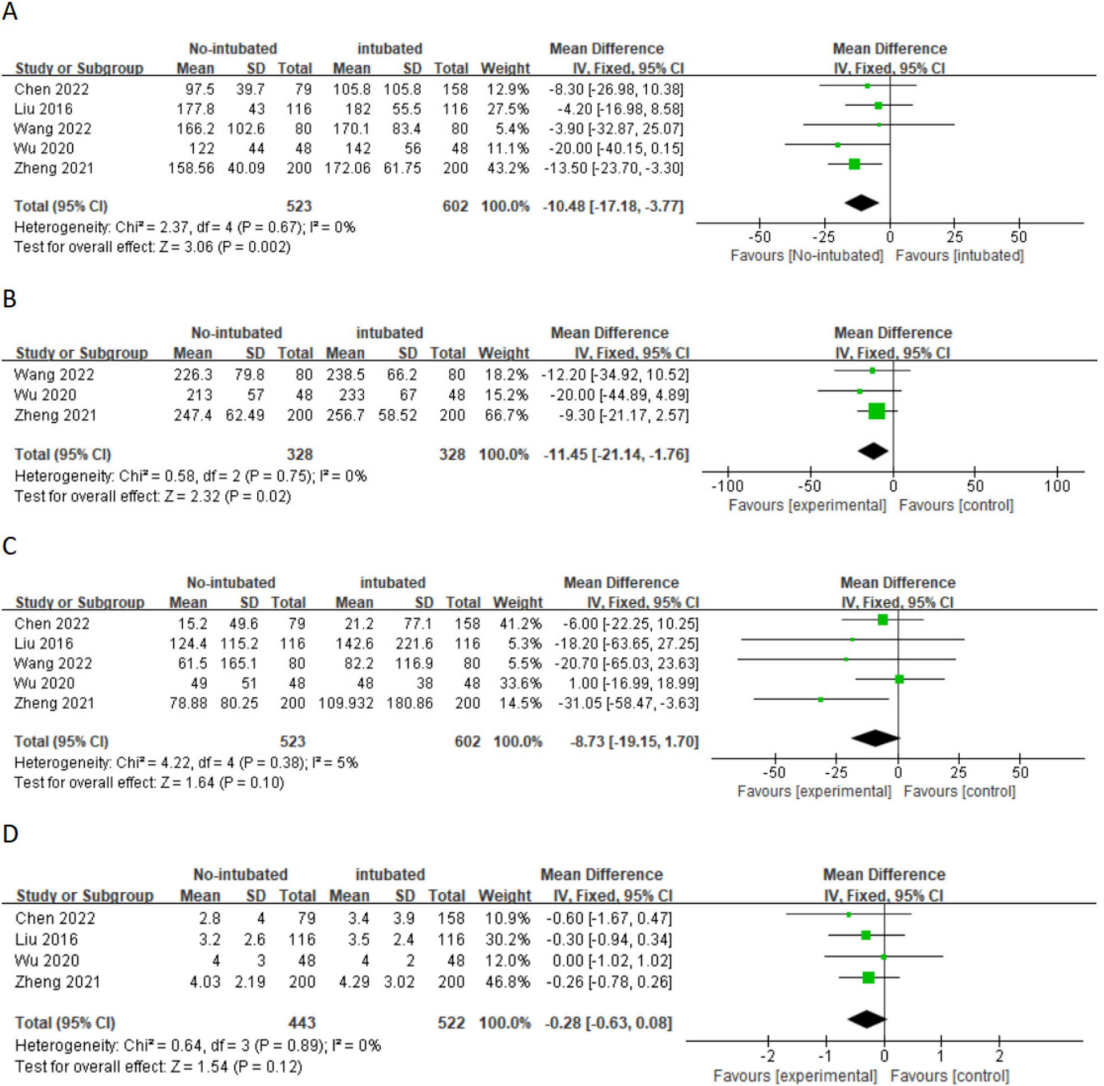
(**A**) Forest plot of operative time comparing the non-intubated group and the intubated group. (**B**) Forest plot of anesthesia time comparing the non-intubated group and the intubated group. (**C**) Forest plot of intraoperative blood loss comparing the non-intubated group and the intubated group. (**D**) Forest plot of chest tube Drainage duration comparing the non-intubated group and the intubated group. CI, confidence interval; IV, inverse variance; SD, standard deviation.

**Figure 6:**
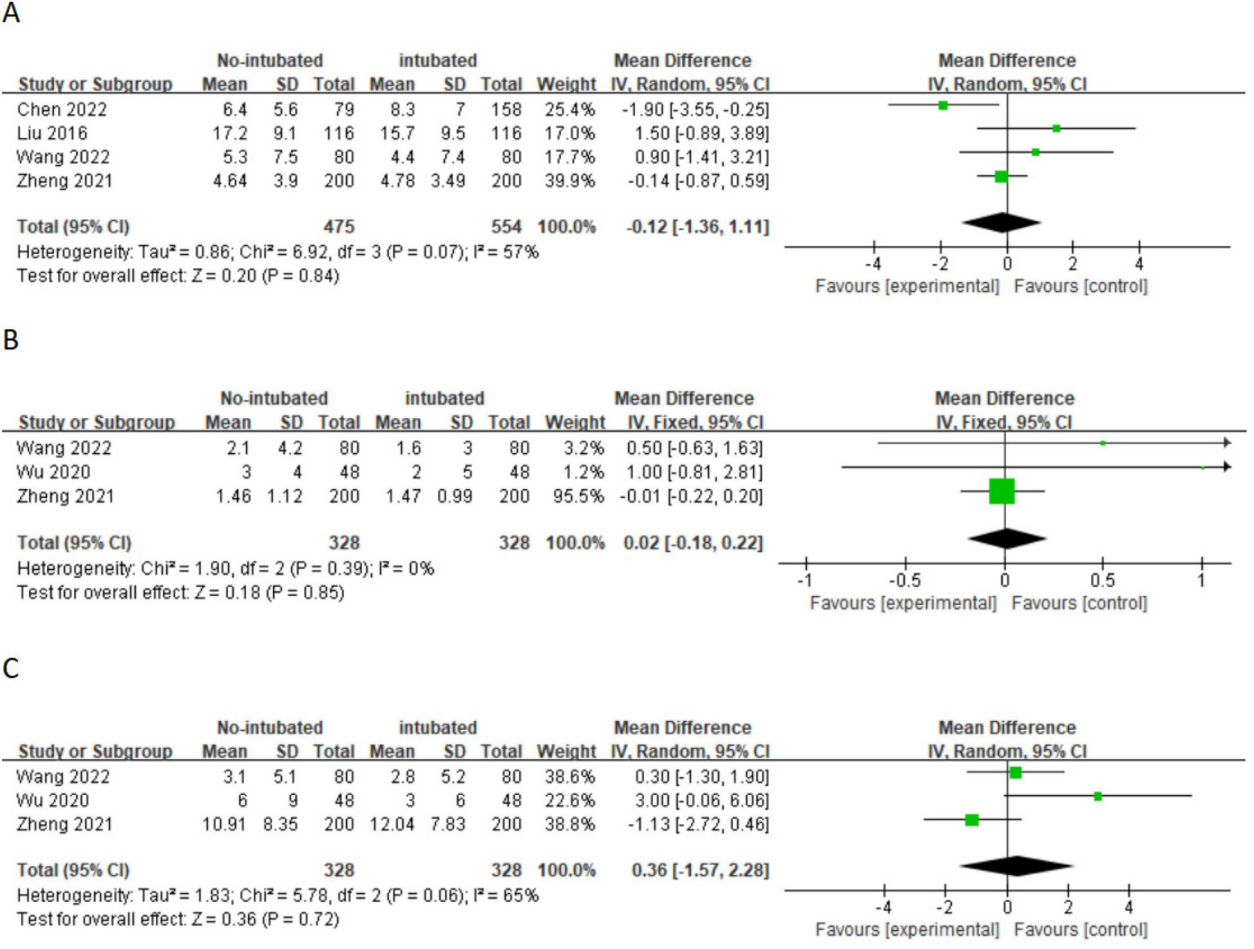
(**A**) Forest plot of lymph node dissection comparing the non-intubated group and the intubated group. (**B**) Forest plot of N1 lymph node dissection comparing the non-intubated group and the intubated group. (**C**) Forest plot of N2 lymph node dissection comparing the non-intubated group and the intubated group.

A subgroup analysis was conducted for patients with non-small cell lung cancer, and the results are shown in Figures 5 and 6 regarding the operation time (WMD = 10.48 min; 95% CI, −17.18 to −3.77; *P* = 0.002) and anesthesia time (WMD = 11.45 min; 95% CI, −21.14 to −1.76; *P* = 0.02). The NIVATS group decreased by approximately 10.48 and 11.45 min, respectively, compared with the IVATS group, and the difference was statistically significant. However, in terms of intraoperative blood loss, thoracic catheter insertion time, and lymph node dissection, the subgroup analysis results indicated that there was no difference between the NIVATS group and the IVATS group.

## Discussion

IVATS under general anesthesia is the standard treatment modality for thoracic surgical procedures. IVATS not only ensures intraoperative oxygenation and facilitates airway management but also provides excellent surgical field exposure. However, some studies have reported that IVATS may lead to airway injuries, barotrauma, dental damage, sore throat, and postoperative muscle weakness associated with the use of muscle relaxants (39, 40). As a novel approach, NIVATS can significantly reduce postoperative pain, lower the incidence of complications, promote patient recovery, and enhance patient comfort. Therefore, NIVATS is gradually being applied in general thoracic surgery. NIVATS typically employs spontaneous ventilation anesthesia, which is generally achieved through a combination of local anesthesia-assisted intravenous anesthesia, including intravenous anesthesia, laryngeal mask airway (LMA), paravertebral blockade, visceral pleural surface anesthesia, and vagus nerve blockade on the surgical side. The avoidance of muscle relaxants helps to prevent delayed postoperative recovery of the cough reflex due to residual muscle relaxants, thereby reducing the risk of postoperative atelectasis and a series of related complications. Despite the numerous advantages of NIVATS over IVATS, there remains controversy regarding whether NIVATS is safer, more effective, and more valuable in the treatment of thoracic diseases. In this study, by limiting the analysis to PSM studies, we have minimized the influence of potential confounding factors present in previously unadjusted observational studies included in earlier network meta-analyses, thereby increasing the confidence in our estimates. In addition, the inclusion of more recent studies allows for a more updated and comprehensive analysis.

This study included a total of 10 studies with 1,882 patients, comparing the safety and efficacy of NIVATS vs. IVATS. Random-effects model analysis showed that NIVATS required shorter operative time and anesthesia time, with statistically significant differences. This is primarily because, first, non-intubated anesthesia requires surgeons to be more focused and meticulous, necessitating the rapid completion of surgery to reduce complications, thereby shortening operative time (41, 42). Second, non-intubated anesthesia no longer uses muscle relaxants, reducing the amount of general anesthesia required and eliminating steps such as intubation, thereby simplifying the anesthesia procedure. The results of this study indicate that there were no significant differences between the NIVATS and IVATS groups in terms of lymph node dissection, N1 lymph node dissection, N2 lymph node dissection, and intraoperative blood loss. Our findings are consistent with those of Wang et al. (30), whereas Chen et al. (32) suggest that during non-intubated surgery, the activation of the cough reflex makes lymph node dissection more difficult. Regarding intraoperative blood loss, Wu et al. (34) suggest that under conditions where patients maintain spontaneous breathing, NIVATS results in more pronounced mediastinal movements, which affect the surgical process and increase technical demands, especially when separating pulmonary vessels and structures, potentially leading to more blood loss in the NIVATS group. These discrepancies indicate that more multicenter, large-sample clinical trials are needed to corroborate these findings. In addition, this study found that NIVATS was associated with a shorter length of hospital stay, whereas there was no significant difference in postoperative length of hospital stay. Length of hospital stay is influenced by multiple factors, including different disease types and the use of antibiotics. Furthermore, patient discharge may depend on the attending physician's subjective assessment of the patient's recovery. Moreover, compared with Western countries, most regions in China lack adequate postoperative local care. Our hospital's patients come from all over the country, and to avoid pleural effusion and readmission, our discharge procedures are relatively conservative. Therefore, further large-scale studies are needed to support the results of this meta-analysis.

Previous studies (43, 44) have shown that tracheal intubation can introduce pharyngeal colonizing bacteria into the lower respiratory tract, leading to symptoms such as respiratory tract infections. In addition, muscle relaxants can significantly increase postoperative nausea and vomiting by reducing intestinal perfusion and oxygen supply, resulting in a higher incidence of postoperative gastrointestinal reactions. Furthermore, residual muscle relaxants can delay the recovery time of patients' ability to cough up sputum after surgery, leading to postoperative pulmonary infections and atelectasis. In contrast to previous studies, our research—which included three related studies—found that the incidence of postoperative complications was higher in the NIVATS group than in the IVATS group. Among them, pulmonary complications were the most common (including atelectasis and pulmonary infection). The incidence of atelectasis in the NIVATS group (19/119) was higher than that in the IVATS group (3/119). As for pulmonary infection, the incidence in the NIVATS group (2/167) was lower than that in the IVATS group (5/167). The main reasons for this result are as follows: First, most patients in the three studies underwent lobectomy, and variations in surgical methods and sites could introduce bias into the results. Second, all three studies were single-center, small-sample, retrospective studies. Third, in the NIVATS group, masks or laryngeal masks were used for lung inflation, whereas in tracheal intubation, the lungs are directly inflated through the endotracheal tube, which may increase the incidence of atelectasis and subsequently lead to a series of pulmonary complications. Moreover, these three studies mainly reported postoperative pulmonary complications and seldom reported complications in other organs, contributing to the observed bias to some extent.

As shown in the above figures, most continuous variables exhibited significant heterogeneity between studies, whereas binary variables did not. Although the surgical method is one of the main reasons for heterogeneity between studies, other factors cannot be excluded, such as differences in medical levels across countries and regions, VATS equipment, research methodologies, and measurement methods. The random-effects model can reduce but not completely eliminate the heterogeneity between studies.

It is particularly important to note that a significant issue with NIVATS is the risk of transitioning from spontaneous ventilation to tracheal intubation. Previous studies have shown that the conversion rate ranges from 0% to 10% (45, 46). Therefore, NIVATS imposes higher technical requirements. First, anesthesiologists must have extensive experience in NIVATS, which specifically includes: (1) proficiency in various thoracic regional block techniques and ensuring their effectiveness is crucial. Inadequate block effect is not the sole cause of intraoperative pain, body movement, coughing, and the need for tracheal intubation. (2) Precise adjustment of sedative and analgesic drug dosages is particularly important. (3) In cases of severe hypoxemia, hypercapnia, uncontrollable coughing/body movement, massive hemorrhage, or circulatory failure, tracheal intubation for airway control and ventilation management must be carried out promptly and decisively, and the conversion must be completed safely within the shortest possible time. Second, diaphragmatic movement and mediastinal swing during spontaneous breathing may affect the surgical field of view, especially during delicate operations such as lymph node dissection and vascular separation. Therefore, surgeons must have extensive experience in thoracoscopic surgery and be capable of performing various thoracoscopic procedures. Lastly, the operation should not be overly long or difficult (47, 48). It is worth noting that NIVATS is more suitable for specific patients with risks of hypoxemia and hypercapnia, for example, (1) partial lung resection, (2) no large areas of adhesion in the lungs, (3) minimal airway secretions, and (4) no contraindications related to epidural anesthesia (49).

This meta-analysis has some limitations. First, all included studies are retrospective; therefore, although propensity score matching was used to reduce selection bias or confounding factors, selection bias cannot be completely excluded. Second, meta-analyses should include more multicenter, large-sample studies; however, most studies included in this research are small-sample, single-center retrospective studies, which may not represent the general situation. Third, some study results indicated high heterogeneity; although we used a random-effects model to reduce heterogeneity, it cannot be completely eliminated, and the small number of included studies prevents subgroup analyses to reduce heterogeneity. Fourth, publication bias (<10 studies for each outcome) cannot be reliably evaluated, and the effect of small-scale studies cannot be excluded. Fifth, the content of the included articles was relatively limited, and some data were missing, so multiple subgroup analyses could not be conducted. Lastly, since this surgery has only been applied clinically in recent years and there are regional differences, the lack of long-term follow-up studies may cause bias in our research results.

## Conclusions

NIVATS is an important development in the field of minimally invasive thoracic surgery. The results of this study further confirm that NIVATS can significantly shorten operative time, anesthesia time, and length of hospital stay. For specific populations, NIVATS may replace IVATS as the primary surgical method. However, the higher incidence of postoperative complications requires further verification through large, well-designed randomized trials.

## Data Availability

The original contributions presented in the study are included in the article/Supplementary Material; further inquiries can be directed to the corresponding author.
